# The Formation and Stabilization of a Novel G-Quadruplex in the 5′-Flanking Region of the Relaxin Gene

**DOI:** 10.1371/journal.pone.0031201

**Published:** 2012-02-21

**Authors:** Sen Lin, Huiping Gu, Ming Xu, Xiaojie Cui, Youyi Zhang, Wei Gao, Gu Yuan

**Affiliations:** 1 Beijing National Laboratory for Molecular Sciences, Key Laboratory of Bioorganic Chemistry and Molecular Engineering of Ministry of Education, Department of Chemical Biology, College of Chemistry and Molecular Engineering, Peking University, Beijing, China; 2 Key Laboratory of Cardiovascular Molecular Biology and Regulatory Peptides of Ministry of Health, Key Laboratory of Molecular Cardiovascular Sciences of Ministry of Education, Institute of Vascular Medicine, Third Hospital, Peking University, Beijing, China; Center for Genomic Regulation, Spain

## Abstract

It has been reported that binding of STAT3 protein to the 5′-flanking region of the relaxin gene may result in downregulation of the relaxin expression. There is a Guanine(G)-rich segment located in about 3.8 Kb upstream of the relaxin gene and very close to the STAT3's binding site. In our study, NMR spectroscopy revealed the formation of G-quadruplex by this G-rich strand, and the result was confirmed by ESI mass spectrometry and CD spectroscopy. The theoretical structure of RLX G-quadruplex was constructed and refined by molecular modeling. When this relaxin G-quadruplex was stabilized by berberine(ΔTm = 10°C), a natural alkaloid from a Chinese herb, the gene expression could be up-regulated in a dose-dependent manner which was proved by luciferase assay. This result is different from the general G-quadruplex function that inhibiting the telomere replication or down-regulating many oncogenes expression. Therefore, our study reported a novel G-quadruplex in the relaxin gene and complemented the regulation mechanism about gene expression by G-quadruplexes.

## Introduction

Relaxin (RLX), a polypeptide hormone, was first discovered in 1926 by Frederick Hisaw [1]. The main functions of relaxin are associated with female reproductive physiology and in particular connective tissue remodeling in the interpubic symphysis and uterine cervix to facilitate birth [1], [2]. In addition to its reproductive associated functions, recent studies revealed diverse actions of relaxin on non-reproductive tissues. Relaxin has been reported to reduce fibrosis in the kidney, heart, lung, and liver [3]. Relaxin could regulate fibroblast proliferation, differentiation, and collagen deposition. Relaxin deficient mice had significantly high left ventricular end-diastolic pressures and age-dependent increasing left ventricular collagen content [4]. Clinical studies also showed increased RLX concentrations positively correlated with severity of congestive heart failure [5].

**Figure 1:**
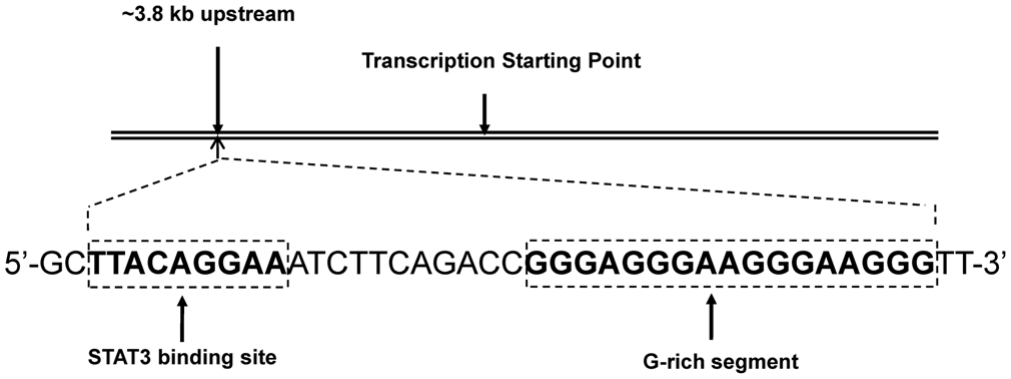
Schematic diagram of the 5′-flanking region of RLX gene.

Relaxin is subjected to many transcriptional factors regulation. Among these factors, STAT3 was considered as the most important one. When STAT3 binds in the 5′UTR of the relaxin gene, it negatively regulates the relaxin expression [6]. G-quadruplex nucleic acids have been reported to be involved in gene expression [7], [8], telomere maintenance [9], [10], [11], [12] and replication stalling [13]. There is a G-rich segment adjacent to the binding site in the 5′-flanking region of the RLX gene (In Figure 1). This G-rich segment has four consecutive G-tracts which have the potential to form a G-quadruplex structure. Our research found that berberine could stabilize the G-quadruplex resulting in up-regulating the expression of the relaxin gene. The elevation of promoter activity might be attributed to the formation and stabilization of the RLX G-quadruplex near STAT3's binding site which could interfere this suppressor's binding to the relaxin gene. The report of this novel discovery will fertilize our knowledge about G-quadruplex functions in cells and provide new clues to develop the G-quadruplex as a target for the therapeutic intervention of relaxin-related diseases.

## Materials and Methods

### Ethics Statement

The investigation conformed to the Guide for the Care and Use of Laboratory Animals published by the US National Institutes of Health (NIH Publication No. 85-23, revised 1996). The experiments were approved by the Committee on Ethics of Animal Experiments and were conducted in accordance with the Guidelines for Animal Experiments, Peking University Health Science Center.

### Oligonucleotides and Natural Product

The oligonucleotides were purchased from Sangon (Shanghai, China). Berberine was purchased from National Institute for the Control of Pharmaceutical and Biological Products (China).

### Mass Spectrometry

ESI mass spectra were obtained in the negative-ion mode with a Finnigan LCQ Deca XP Plus ion-trap mass spectrometer (San Jose, CA). The direct-infusion flow rate was 2.0 µL/min. The electrospray-source conditions were 2.7 kV spray voltage and 120°C capillary temperature.

### Circular Dichroism

The CD spectra of DNA were measured by using a J-810 spectropolarimeter (JASCO Co., Ltd., Japan) with a 0.1 cm path-length quartz cell at 25°C. All the samples were annealed at 90°C for 10 min and then cooled to room temperature overnight.

### Thermal Denaturation

Melting curves were obtained by monitoring the changing at a wavelength 264 nm which has the highest CD value. The CD spectra were measured by using a J-810 spectropolarimeter (JASCO Co., Ltd., Japan) with a 0.1 cm path-length quartz cell. The procedures to analyze the thermodynamic parameters were included in Text S1.

### NMR Titrations

The strand concentration of NMR samples was typically 0.81–1.31 mM; the solutions contained potassium phosphate (pH 7.0) as buffer, as well as 10% D_2_O. 5 mM Berberine solution was titrated into the sample solution to achieve different molar ratios. All experiments were performed on 600 MHz Bruker spectrometers at 298 K.

### Molecular Modeling

The solution structure of quadruplex in human c-MYC promoter (PDB code 1xav) [14] was used as an initial model and then treated by some necessary replacement and deletion to construct the RLX G-quadruplex structure. Each system was neutralized by Na^+^ and solvated with a truncated octahedral box containing an 8 angstrom buffer of TIP3P water. Energy minimization was performed in these explicit solvents using the sander module of AMBER 10 [15], [16]. First, the system of G-quadruplex soaked in water was minimized for 1000 steps with holding the DNA fixed by 500 kcal/mol^−1^Å^−2^. Then 2500 steps of minimization were done for the entire system with no restraints. This was followed by molecular dynamics with 20 ps equilibration while restraining the solute by 10 kcal/mol^−1^Å^−2^ and then 20 ns simulations for the whole system at 300 K. Trajectories with constant RMSD were averaged using the ptraj module and the obtained structures were finally minimized in explicit system. Berberine was docked with the G-quadruplexes by the software AutoDock3.0 [17], [18], [19]. Genetic algorithm was used as the search method in docking. All the default parameters were chosen provided by the software.

### Cell culture

Cardiac fibroblasts were isolated and cultured from 1 day-old neonatal SD rats as described previously [20], [21]. All experiments were performed in fibroblasts at passage 2–3. The purity of these cultures was greater than 98% cardiac fibroblasts, as determined by positive staining for vimentin and negative staining for von Willebrand factor. Studies involved cardiac fibroblasts grown to 80% confluence and serum starved for 24 h in serum-free medium before treatment.

### Luciferase assay

The pGL3-basic vector was obtained from Promega. A 4.5-kb fragment of rat RLX promoter was generated by PCR using genomic DNA isolated from cardiac fibroblasts as a template. The DNA was purified by using the Qiagen DNA Purification Kit. The RLX promoter PCR primers included a forward primer starting at bp 4117 (relative to the start of the first exon), a reverse primer starting at bp +382. The amplified fragments were attached to Hind III- and Kpn I-digestion sites of the pGL3-basic vector. The pGL3 vectors harboring ligation products were introduced into E. coli. Fifty microliters of each transformed-bacteria batch was spread onto agar plates containing ampicillin and incubated overnight at 37°C. Single ampicillin-resistant colonies were microaspirated and analyzed following restriction enzyme digestion. The sequences of all constructs were verified by automated sequencing. The pGL3 basic reporter constructed 4.5-kb rat RLX promoter was used for site-specific mutation (gggagggaagggaaggg→gtgagtgaagtgaagtg).

To testify the effect of berberine on RLX promoter activity HEK293 cells were used. HEK293 in 24-well plates were cotransfected with luciferase constructed or empty pGL3 vector (400 ng) and the internal control p-RL-TK vector (20 ng) using lipofectamine 2000 for 24 hours. Cells were harvested and luciferase activity was measured using the Dual-Luciferase Reporter Assay System according to the manufacturer's instructions. The relative activity was normalized by the ratio of firefly luciferase activity to Renilla luciferase activity and calculated as the folds to control pGL3-basic or pcDNA3 empty vector.

## Results and Discussion

### The formation of RLX G-quadruplex

**Figure 2:**
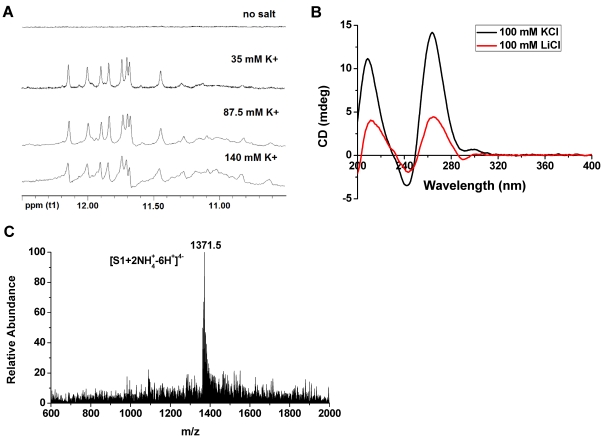
Formation of RLX G-quadruplex. (A) ^1^H-NMR titration of G-rich sequence S1 with KCl. Increasing amounts of the K^+^ were serially added to the DNA solution as indicated on each scan. The titration was carried out at 298 K and used K_2_HPO_4_-KH_2_PO_4_ (pH = 7.0) as buffer solution; (B) CD spectra of 10 µM S1 in the presence of 100 mM KCl or LiCl (30 mM Tris-HCl, pH = 7.4); (C) ESI mass spectrum of 10 µM S1 in 100 mM NH_4_Ac (pH = 7.0) and 20% methanol solution.

The RLX G-quadruplex formation from the G-rich oligonucleotide (S1, 5′-GGGAGGGAAGGGAAGGG-3′) was probed by NMR spectroscopy. In the proton NMR spectrum (Figure 2A), there was no signal between 10.5–12.5 ppm in 10% D_2_O, but the signals emerged after titrating K^+^ into the DNA solution. These NMR signals in the region 10.5–12.5 ppm belongs to the imino protons of G-quartet [22], [23], [24], [25], [26], [27], this result indicated that the addition of K^+^ ion could induce the formation of the G-quadruplex structure by the strand S1. When this G-rich oligonucleotide S1 was dissolved in 100 mM KCl (30 mM Tris-HCl, pH = 7.4), the CD spectrum (Figure 2B) showed a strong positive peak at 264 nm and a negative peak at 242 nm which confirmed the formation of a parallel strand of G-quadruplex at near physiological conditions [28], [29]. But the intensity of the maximum peak at 264 nm in CD spectra was significantly decreased in the presence of 100 LiCl (Figure 2B). The ESI-MS spectrum of this DNA solution S1 [methanol∶water = 20∶100( v/v)] showed that, in Figure 2C, at 100 mM ammonium acetate, the intensities of the complex ion for the G-quadruplex ion with two NH_4_^+^, [S1+2NH_4_^+^-6H^+^]^4−^, became the base peak (100%). Since NH_4_^+^ is known to sit between two G-tetrad layers, it would be reasonable that there are three tetrad layers in the RLX G-quadruplex DNA possessing two NH_4_^+^ ions between them [30], [31]. The result from ESI mass spectra provided once more supports for the formation of RLX G-quadruplex accompanied by cations.

**Figure 3:**
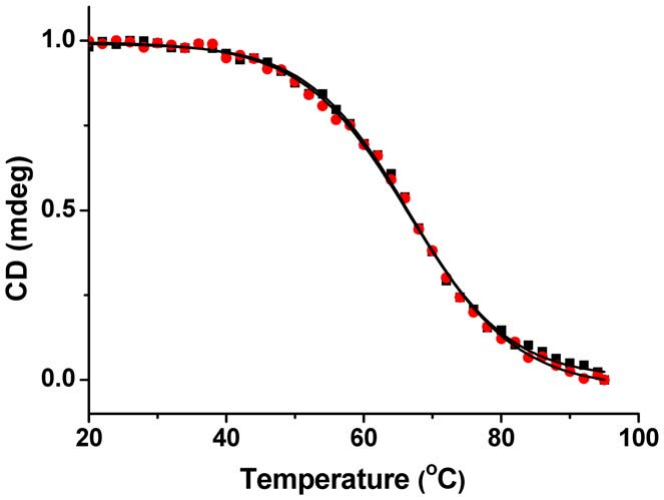
CD Tm of RLX G-quadruplex. Normalized CD melting temperature curves of 10 µM (red circle) and 20 µM (black square) RLX G-quadruplex (S1) at 264 nm in 30 mM Tris-HCl buffer (pH = 7.4) containing 100 mM KCl.

The CD melting temperature (Tm) of this RLX G-quadruplex at 264 nm was 67°C in 100 mM KCl and 30 mM Tris-HCl (pH = 7.4) buffer condition. When the strand concentration of RLX G-quadruplex (S1) was changed from 10 µM to 20 µM, its CD Tm stayed the same (Figure 3), which indicated that it is an intramolecular G-quadruplex. But when varying different cations, its thermal stability would be decreased which was 37°C in 100 mM LiCl solution (Figure S3). According to the analysis above, this G-rich sequence S1 in the 5′-flanking region of RLX gene can form a stable intramolecular G-quadruplex at near physiological conditions.

### The stabilization of RLX G-quadruplex

**Figure 4:**
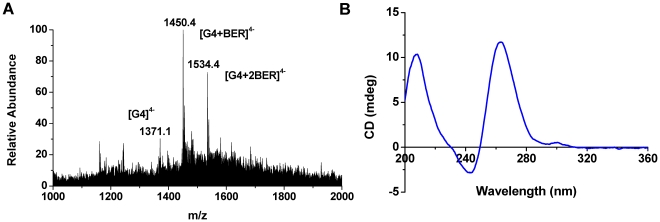
Complex of RLX G-quadruplex and berberine. (A) ESI mass spectrum (100 mM NH_4_Ac, pH = 7.0) and (B) CD spectrum (100 mM KCl, 30 mM Tris-HCl, pH = 7.4) for the complex of 10 µM RLX G-quadruplex (G4) and 40 µM berberine at a molar ratio 1∶4.

In order to find RLX G-quadruplex ligands, many small molecules, such as dehydrocorydaline [31], were chosen as recognizing-molecule candidates. The results showed that berberine (**BER**, Scheme S1), a natural alkaloid which has been used as an antidiarrheal agent in Chinese traditional medicine and reported to be able to target telomeric G-quadruplex [32], [33], [34], [35], had a very good binding with this RLX G-quadruplex. As shown in Figure 4A, when berberine was added into the RLX G-quadruplex solution at a concentration ratio of 1∶4, the intensity of the 1∶1 complex ion [RLX-G4+**BER**]^4−^ at m/z 1450.4 was 100% (base peak) and the intensity of 1∶2 complex ion [RLX-G4+2**BER**]^4−^ at m/z 1534.4 was 75% in ESI mass spectrum, while the intensity of free G-quadruplex ion [RLX-G4]^4−^ was only 30%. Here, a parameter IR_a_ (Scheme S2) is used for analyzing the relative binding affinity of berberine with RLX G-quadruplex [31].

In the formula, IR_a_ (0<IR_a_<1.0) is the intensity ratio of all complex ions to the sum of all G-quadruplex and complex ions in an ESI mass spectrum. ∑I_r_(G), ∑I_r_(G+P_i_), ∑I_r_(G+2P_i_) and ∑I_r_(G+3P_i_) are the total intensities of G-quadruplexes (G), 1∶1, 1∶2 and 1∶3 complex ions, respectively. The IR_a_ value for berberine binding with RLX G-quadruplex was calculated to be 0.85 according to the mass spectrum in Figure 4A.

**Figure 5:**
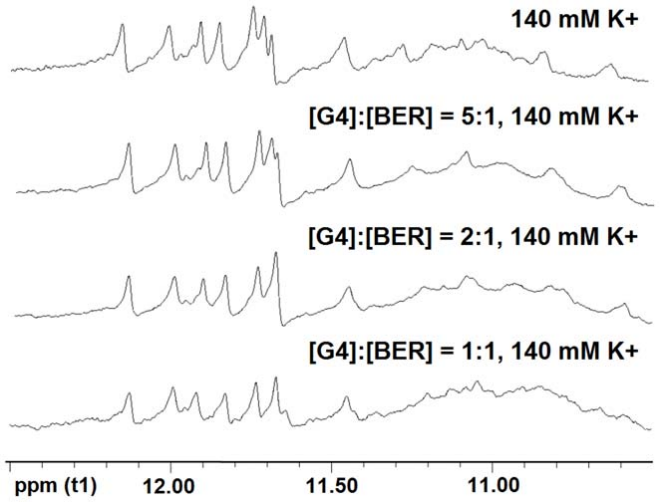
^1^H-NMR titration of RLX G-quadruplex (G4) with berberine. Increasing amounts of the berberine were serially added to the DNA solution as indicated on each scan. The titration was carried out at 298 K and used K_2_HPO_4_-KH_2_PO_4_ (pH = 7.0) as buffer solution.

In CD spectrum of the complex of berberine with the RLX G-quadruplex (Figure 4B), the characteristic peak at 264 nm clearly presented that the complex still kept the parallel strand orientation after the binding. ^1^H-NMR titration research (Figure 5) showed that the peaks of the RLX G-quadruplex from 10.5 to 12.5 ppm were shifted when berberine was titrated into the RLX G-quadruplex solution. This proved once again that Berberine could interact with the RLX G-quadruplex.

**Figure 6:**
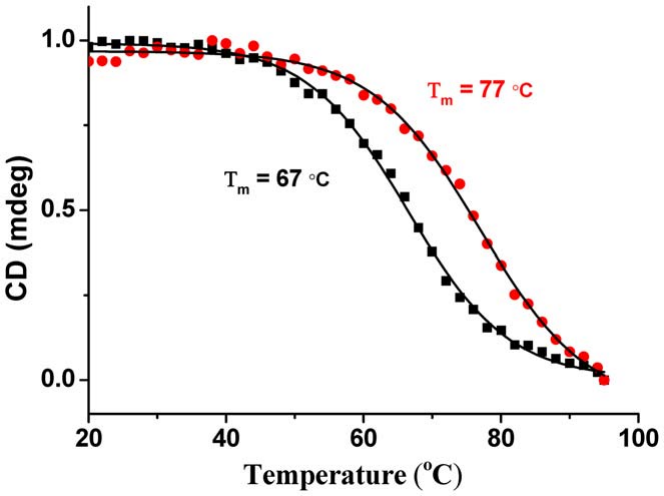
CD Tm of complex of RLX G-quadruplex and berberine. Normalized CD melting temperature curves at 263.8 nm for 10 µM RLX G-quadruplex (S1) before (black square) and after (red circle) adding 40 µM berberine (100 mM KCl, 30 mM Tris-HCl buffer, pH 7.4).

More important, the CD temperature melting experiment showed that the Tm value of 10 µM RLX G-quadruplex in 100 mM KCl (30 mM Tris-HCl buffer) was increased from 67°C to 77°C after adding 40 µM berberine into the solution (Figure 6). We probed the thermal melting profile of berberine binding to RLX G-quadruplex by van't Hoff's method [36] using procedures described by Mergny [37] and Chaires [38]. The berberine binding was enthalpically favored compared to DNA melting alone by −2090.8 J/mol, meanwhile the Gibbs free energy for the RLX G-quadruplex was favored by −11.0 kJ/mol at 67°C (shown in Table 1).

**Table 1:** Thermodynamic parameters of RLX G-quadruplex.

Ligand	Δ*H* (J/mol)	Δ*S* (J/mol)	Δ*G* (kJ/mol) at 67°C
None	−2110.2	35.3	−14.1
**BER** (1∶4)	−4201.0	61.4	−25.1

10 µM RLX G-quadruplex in 100 mM KCl with or without **BER** (30 mM Tris-HCl buffer, pH 7.4).

The results above conclude that the RLX G-quadruplex can be stabilized by berberine. Therefore berberine provides a good molecular skeleton for the drug development to target RLX G-quadruplex and for the exploring the novel functions of the RLX G-quadruplex in cell.

### The structure of RLX G-quadruplex

**Figure 7:**
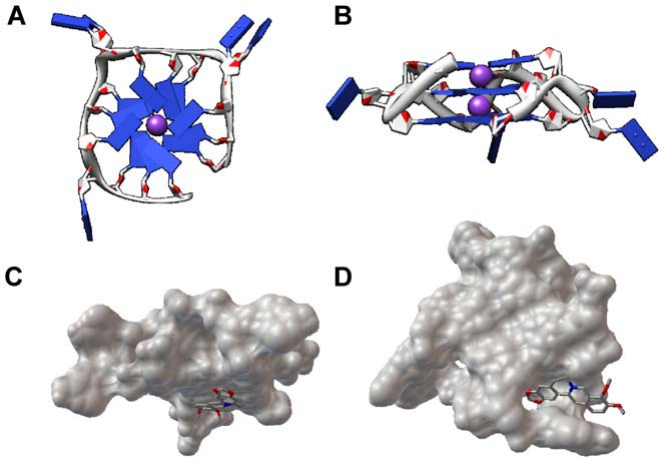
RLX G-quadruplex structure models presented by software Chimera [**41**]. (A) Top view (B) side view. (The purple balls stand for potassium ions). The docked results of berberine with RLX G-quadruplex presented by software PMV [42] (C) end stacking (D) groove binding. (Berberine was shown as stick model while the G-quadruplex as molecular surface model).

There are only three guanines in each G-rich track of S1, which means that it can form a G-quadruplex with three G-quartet layers [39], [40]. The theoretical structure of RLX G-quadruplex was constructed through treating a solution G-quadruplex structure (PDB code 1xav) [14] by some necessary replacement and deletion and then refined through 20 ns molecular dynamics simulation. The structures models are shown in Figure 7A and 7B. We can see that two potassium ions are intercalated between the three G-quartet layers and all the bases in the loop region are pointing far out to make the structure much more stable. Molecular docking was further done to give an insight into the interaction between the ligand berberine and RLX G-quadruplex. According to the docking results, the binding modes are mainly end stacking and groove binding. In Figure 7C, berberine would interact along the G-quartet surface to get an end stacking binding mode. However, berberine could also fix the binding pocket very well in the G-quadruplex groove interacting with the bases of loop regions (Figure 7D). A lot of noncovalent bonds gave contributions to the high binding affinity between them. Especially the π-π stacking, between the aromatic function groups of berberine and the base-surface of the RLX G-quadruplex, was the dominant contribution for their tight binding. It is worth noting that, in Scheme S1, berberine owns a large aromatic-planar skeleton, which means it is much easier to fit the binding sites of RLX G-quadruplex forming more π-π stacking contacts. To some extent, these two binding modes explained how one RLX G-quadruplex was able to bind with two berberine molecules as shown by the mass spectrum in Figure 4A.

### Formation of RLX G-quadruplex and stabilization by berberine could up-regulate RLX gene expression

**Figure 8:**
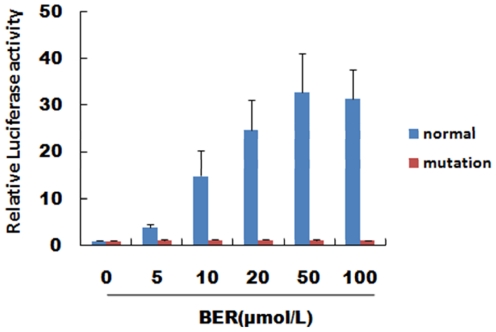
Luciferase assay. Results demonstrated that the formation and stabilization of G-quadruplex induced by berberine could increase RLX promoter activity about 10∼40 folds and in a dose-dependent manner (blue column). Berberine has no effect on RLX promoter activity when the G-rich region mutated (red column). Results are presented three to four separate experiments.

To testify the effect of berberine on RLX, luciferase assay was used. HEK293 in 24-well plates were cotransfected with luciferase constructed or empty pGL3 vector as well as the internal control p-RL-TK vector for 24 hours. To investigate the role of G-quadruplex on RLX activity, we designed another RLX vector which was site-specific mutated (gggagggaagggaaggg→gtgagtgaagtgaagtg). The mutation of guanines in the G-tracks will disrupt the formation of G-quadruplex or promote the presence of another conformation [43], [44]. In general, the characteristic positive maximum peak in CD spectrum for parallel G-quadruplex is at 264 nm and for the anti-parallel one is at 295 nm [28], [29]. RLX native G-rich sequence (5′-GGGAGGGAAGGGAAGGG-3′) showed a positive maximum at 264 nm which indicates the formation of a G-quadruplex with parallel strand orientation. However, in Figure S1, the mutated G-rich sequence (5′-GTGAGTGAAGTGAAGTG-3′) showed a positive maximum at 272 nm in the same buffer solution. Moreover, compared with the native sequence (Figure 2B), the control sequence did not show a significant transition of the maximum peak intensity when changing the buffer solution from 100 mM LiCl to 100 mM KCl (Figure S1). It suggested that the mutation one promoted another conformation of DNA not a typical parallel G-quadruplex [45]. When we mixed RLX G-quadruplex and berberine at a ratio of 1∶4, the complex ion became the base peak in the ESI mass spectrum (Figure 4A). But when it comes to the mutated sequence with berberine, the base peak was still the DNA's multi-charge ion, without any dominant complex ion, even at a ratio of 1∶4 in the same buffer condition (Figure S2). The results presented that the mutated one of the RLX G-rich sequence did not have a good affinity with berberine because it could not form a typical parallel G-quadruplex structure. So luciferase assay in Figure 8 demonstrated that the formation and stabilization of RLX G-quadruplex by berberine could up-regulate gene expression in a dose-dependent manner. (The raw data of luciferase activity at BER 0 µmol/L is provided in Figure S4)

In conclusion, the present study has revealed the formation of a G-quadruplex by the G-rich strand in the 5′-flanking region of the RLX gene by NMR spectroscopy and this result was confirmed by ESI mass spectrometry and CD spectroscopy. The theoretical structure of RLX G-quadruplex was constructed and refined by molecular modeling. Luciferase assay demonstrated that the formation and stability of the G-quadruplex could increase the gene expression. This work will complement the G-quadruplex functions about gene regulation.

## Supporting Information

Text S1 **Analysis of thermodynamic parameters using van't Hoffs method.**
(DOC)

Scheme S1 **Structure of Berberine (BER).**
(TIFF)

Scheme S2 **Formula of parameter IR_a_.**
(TIFF)

Figure S1 **CD spectra of RLX mutated sequence.** 10 µM control sequence RLX-TTTT (5′-GTGAGTGAAGT GAAGTG-3′) in the presence of 100 mM KCl or LiCl (30 mM Tris-HCl, pH = 7.4);
(TIFF)

Figure S2 **ESI mass spectrum the complex of RLX mutated sequence.** 10 µM RLX-TTTT (5′-GTGAGTGAAGTGAAGTG-3′) and 40 µM berberine at a ratio of 1∶4 (100 mM NH_4_Ac, pH = 7.0).
(TIFF)

Figure S3 **CD Tm of RLX G-quadruplex in LiCl.** CD melting temperature curve of 10 µM RLX G-quadruplex (S1) at 264 nm in 30 mM Tris-HCl buffer (pH = 7.4) containing 100 mM LiCl.
(TIFF)

Figure S4 **Luciferase assay (Raw data).** Results demonstrated that the formation of RLX G-quadruplex could increase transcriptional activity (normal sequence, …GGGAGGGAAGGGAAGGG…; mutation one, …GTGAGTGAAGTGAAGTG…).
(TIFF)
