# Supplementary material for: The Formation and Stabilization of a Novel G-Quadruplex in the 5′-Flanking Region of the Relaxin Gene
Source: PLoS One. 2012 Feb 21;7(2):e31201. doi: 10.1371/journal.pone.0031201 (PMC3283602; doi:10.1371/journal.pone.0031201)
Supplement: Text S1 — Analysis of thermodynamic parameters using van't Hoffs method. (DOC) [file pone.0031201.s001.doc]

**Analysis of thermodynamic parameters using van’t Hoffs method.**

The melting curves were plotted in Origin 8.0 and the upper and lower baselines were linearly fit. Next the fraction folded parameter θ(T) was computed for each temperature (T) according to Eq. 1.

$\theta=\frac{A_{U}(T)-A(T)}{A_{U}(T)-A_{L}(T)}=\frac{m_{U}T+b_{U}-A(T)}{(m_{U}-m_{L})T+b_{U}-b_{L}}$ (1)

*θ* = fraction folded parameter, *A(T)* = absorbance at temperature T, *m* = slope, *b* = intercept, *U* stands for the upper baseline and *L* for the lower baseline.

Since this quadruplex is an intramolecular one, the data was then converted into yield association constants *K_α_* using Eq. 2.

$K_{\alpha}=\frac{\theta}{1-\theta}$ (2)

Note that the data in the range 0.15 < *θ* < 0.85 was used in order to keep *K_α_* most precise. By definition, the free Gibbs enthalpy could be written as

$\Delta G=-RT\ln(K_{\alpha})=\Delta H-T\Delta S$ (3)

Then ln(*K_α_*) was plotted versus 1/T to afford a linear relationship which gave –△H/R as slope and △S/R as intercept.

1. Ranjan, N., Andreasen, K.F., Kumar, S., Hyde-Volpe, D. and Arya, D.P. (2010) Aminoglycoside Binding to Oxytricha nova Telomeric DNA. *Biochemistry-Us*, **49**, 9891-9903.

2. Mergny, J.L. and Lacroix, L. (2003) Analysis of thermal melting curves. *Oligonucleotides*, **13**, 515-537.
