# Supplementary figures and images for: The Formation and Stabilization of a Novel G-Quadruplex in the 5′-Flanking Region of the Relaxin Gene
Source: PLoS One. 2012 Feb 21;7(2):e31201. doi: 10.1371/journal.pone.0031201 (PMC3283602; doi:10.1371/journal.pone.0031201)

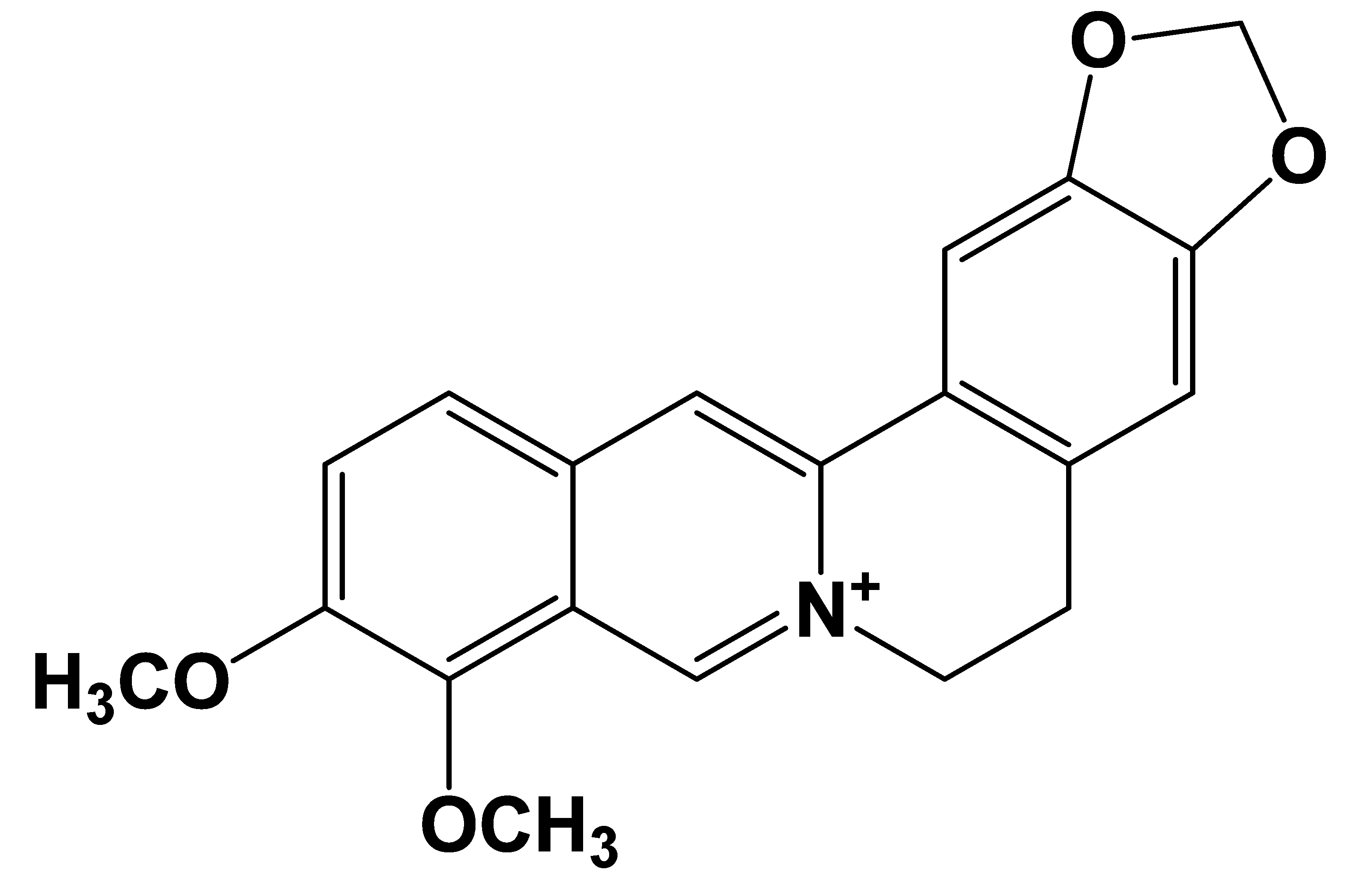

Supplement: Scheme S1 — Structure of Berberine (BER). (TIFF) [file pone.0031201.s002.tiff]

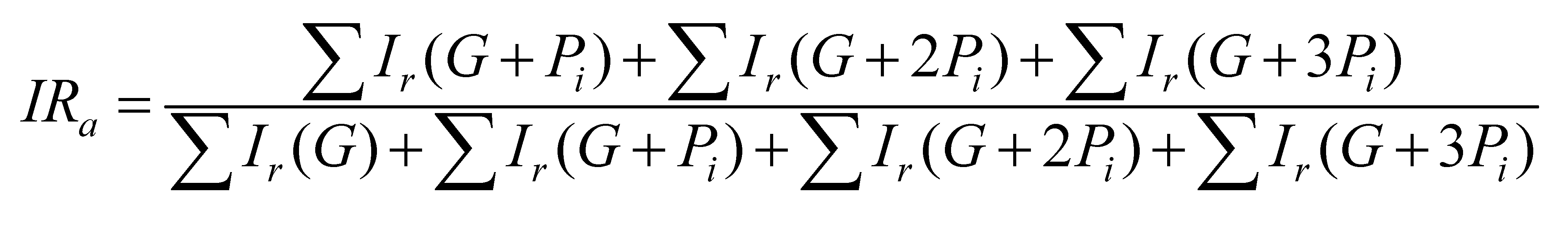

Supplement: Scheme S2 — Formula of parameter IRa. (TIFF) [file pone.0031201.s003.tiff]

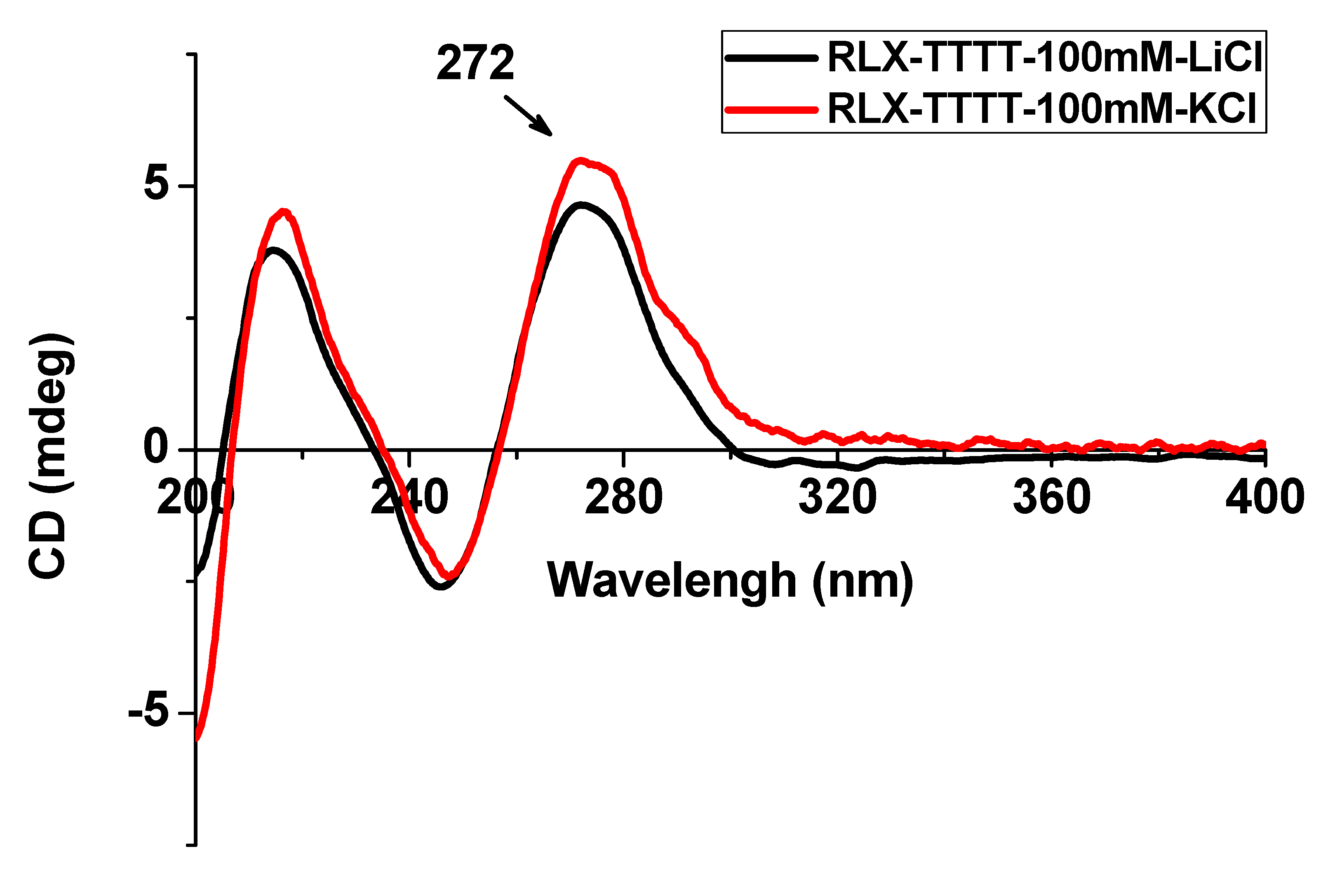

Supplement: Figure S1 — CD spectra of RLX mutated sequence. 10 µM control sequence RLX-TTTT (5′-GTGAGTGAAGT GAAGTG-3′) in the presence of 100 mM KCl or LiCl (30 mM Tris-HCl, pH = 7.4); (TIFF) [file pone.0031201.s004.tiff]

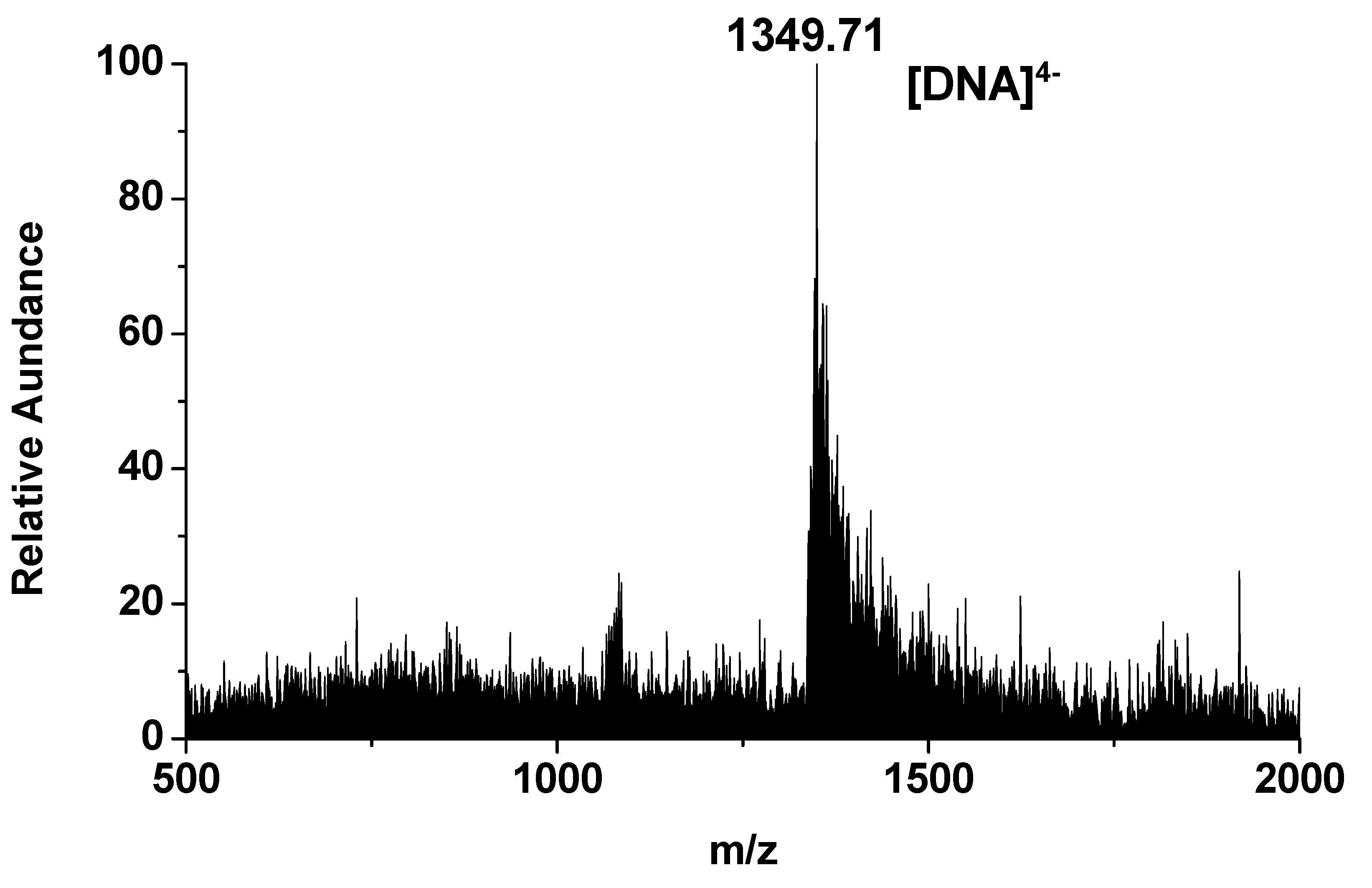

Supplement: Figure S2 — ESI mass spectrum the complex of RLX mutated sequence. 10 µM RLX-TTTT (5′-GTGAGTGAAGTGAAGTG-3′) and 40 µM berberine at a ratio of 1∶4 (100 mM NH4Ac, pH = 7.0). (TIFF) [file pone.0031201.s005.tiff]

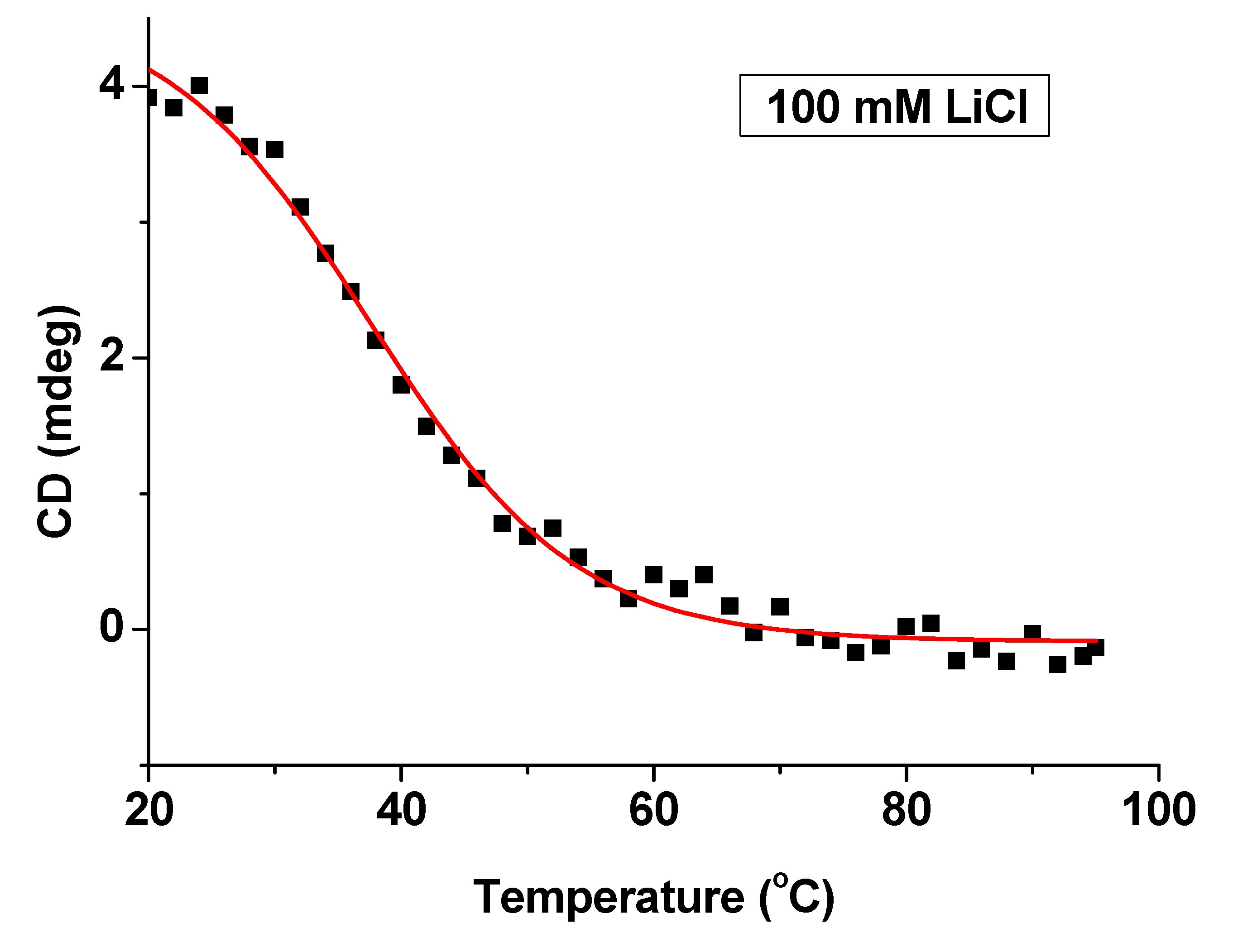

Supplement: Figure S3 — CD Tm of RLX G-quadruplex in LiCl. CD melting temperature curve of 10 µM RLX G-quadruplex (S1) at 264 nm in 30 mM Tris-HCl buffer (pH = 7.4) containing 100 mM LiCl. (TIFF) [file pone.0031201.s006.tiff]

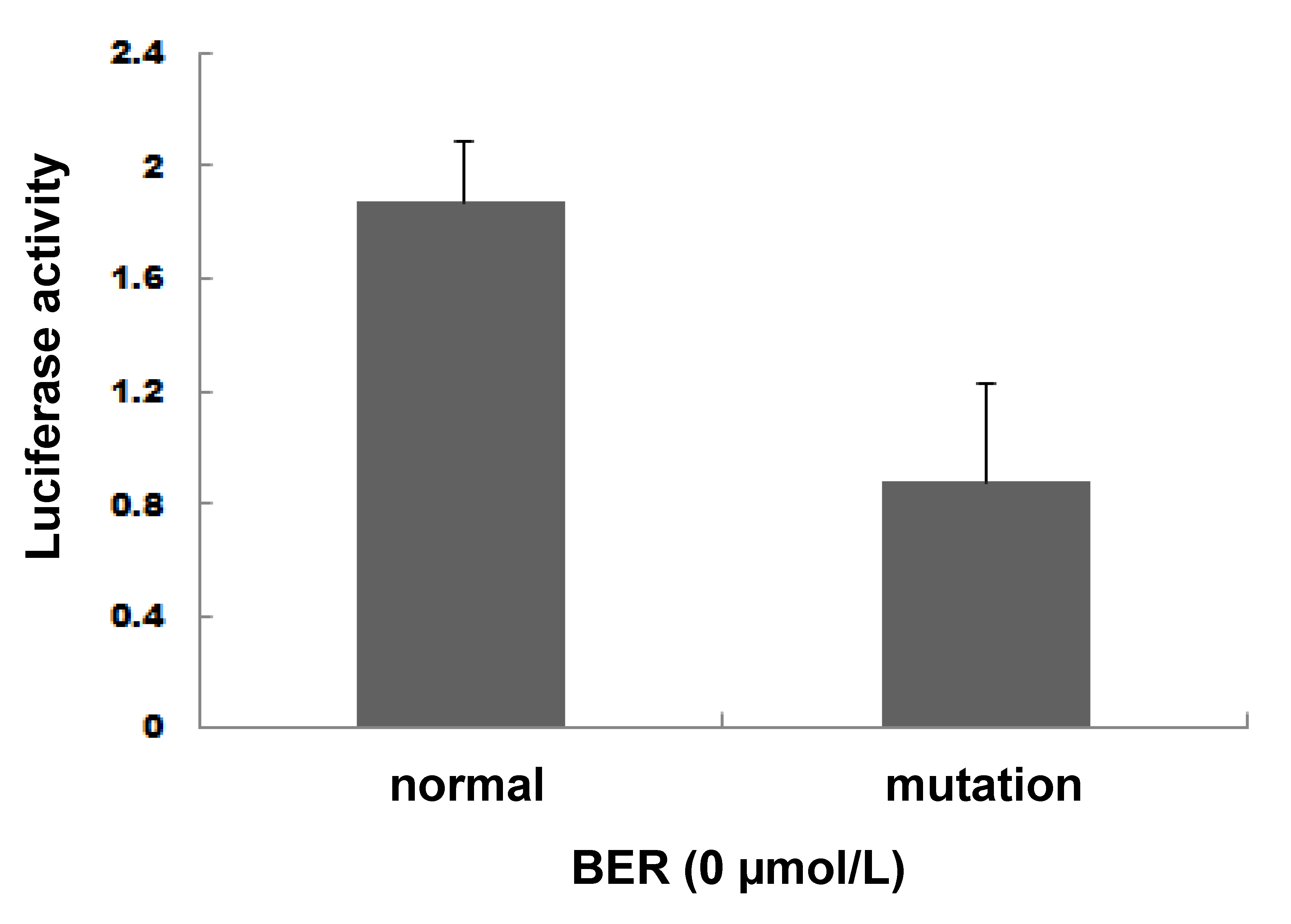

Supplement: Figure S4 — Luciferase assay (Raw data). Results demonstrated that the formation of RLX G-quadruplex could increase transcriptional activity (normal sequence, …GGGAGGGAAGGGAAGGG…; mutation one, …GTGAGTGAAGTGAAGTG…). (TIFF) [file pone.0031201.s007.tiff]
